# Immune Checkpoint Inhibitor-Induced Psoriasiform, Spongiotic, and Lichenoid Dermatitis: A Novel Clinicopathological Pattern

**DOI:** 10.7759/cureus.28010

**Published:** 2022-08-14

**Authors:** Yana Kost, Daiva Mattis, Ahava Muskat, Bijal Amin, Beth McLellan

**Affiliations:** 1 Dermatology, Albert Einstein College of Medicine, Bronx, USA

**Keywords:** oncodermatology, interleukin 17, spongiotic, lichenoid, psoriasiform, immune-related adverse event, immune checkpoint inhibitor

## Abstract

Immune checkpoint inhibitors (ICIs), a class of anticancer agents that upregulate T-cell response to tumor cells, are associated with immune-related adverse events (irAEs), and the skin is one of the most commonly affected organs. We report the first two cases of a unique ICI-induced clinicopathological entity. A psoriasiform-appearing eruption with psoriasiform, spongiotic, and lichenoid dermatitis pattern on histopathology. A 73-year-old male with stage IV melanoma treated with nivolumab and a 63-year-old female with stage IV colorectal cancer treated with pembrolizumab and TAK-981 separately presented to our clinic with a psoriasiform rash. In both patients, punch biopsy revealed an unusual combination of psoriasiform, spongiotic, and lichenoid dermatitis. Treatment with apremilast in the first patient yielded some improvement, while treatment with ixekizumab in the second patient yielded a complete resolution of the eruption. Our cases add to the growing body of reported immune toxicities related to ICI use and illustrate the utility of targeted immune suppression of pathways in disease phenotype to allow for ICI continuation and optimization of cancer treatment.

## Introduction

Recent advances in anticancer therapeutics significantly improve patient prognosis. However, a wide variety of dermatologic toxicities have developed in parallel, detrimental to patients’ quality of life and may limit cancer treatment. Immune checkpoint inhibitors (ICIs), a class of anticancer agents that upregulate T-cell response to tumor cells, cause approximately half of the patients to experience immune-related adverse events (irAEs) [1]. Notably, cutaneous irAEs are the most common [1]. We report the first two cases of a unique ICI-induced clinicopathological entity, a psoriasiform-appearing eruption with psoriasiform, spongiotic, and lichenoid dermatitis pattern on histopathology.

## Case presentation

Case 1

A 73-year-old male with stage IV melanoma and complex dermatologic history of several irAEs, including Grover's disease of the trunk, eruptive keratoacanthomas, and inflamed seborrheic keratoses, presented with a one-month history of pruritic rash on the arms and legs. The rash began 15 months after starting 480 mg adjuvant nivolumab therapy IV for a brain metastasis following seven years of stable disease after excision for a stage 1B scalp melanoma lesion. At the time of presentation, the patient was receiving treatment with 300 mg dupilumab, 5 mg prednisone, and 100 mg doxycycline for Grover's disease.

**Figure 1:**
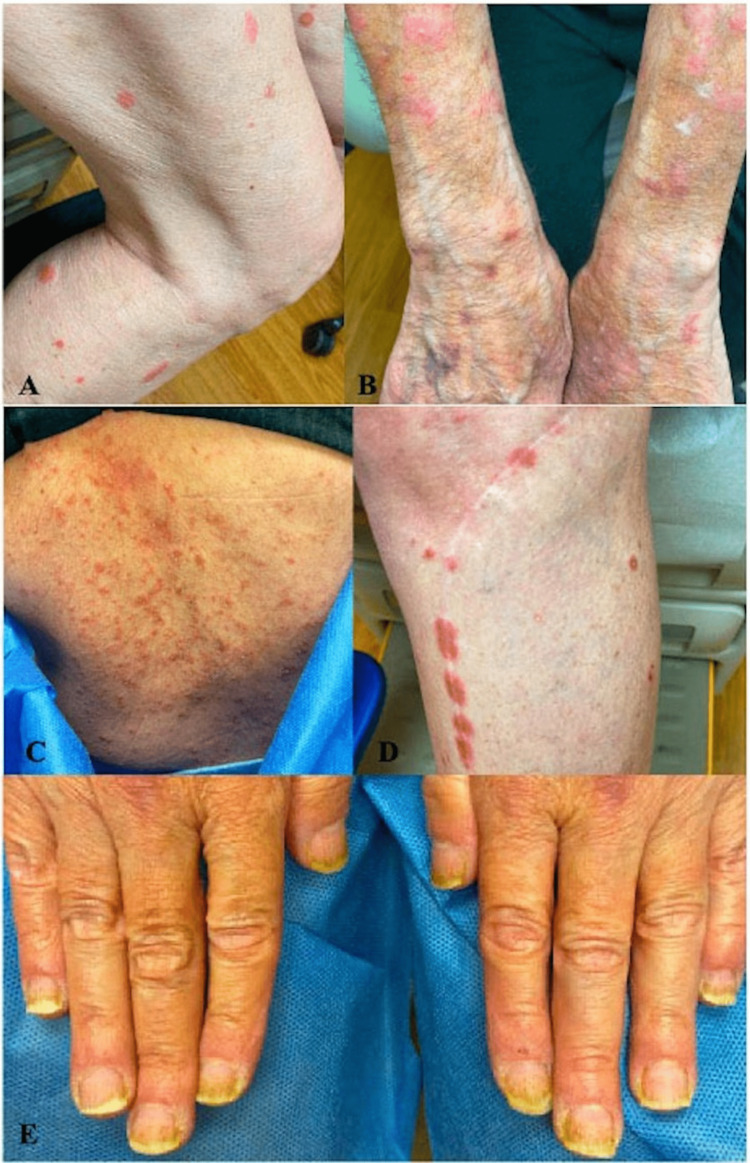
Psoriasiform rash. (A) The patient's legs demonstrating discrete, pink, scaly plaques. (B) The same lesions shown on the patient's arm after one-month-long course of treatment with acitretin. (C) The patient's back demonstrating diffuse discrete, pink, scaly macules and plaques. (D) The patient's knee demonstrating koebnerization of pink, scaly plaques localized to a previous scar. (E) Bilateral distal nail yellowing and splinter hemorrhages.

**Figure 2:**
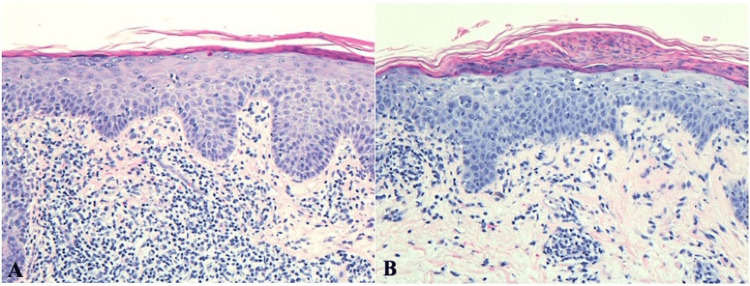
Psoriasiform, spongiotic, and lichenoid dermatitis. (A) Punch biopsy obtained from the right anterior thigh of the patient in case 1. (B) Punch biopsy obtained from the left lower back of the patient in case 2. Both demonstrate parakeratosis, psoriasiform hyperplasia, spongiosis, and lichenoid dermatitis. Both also show rare necrotic keratinocytes in the upper epidermis and purpura in the dermis (H&E, 200x).

On examination, discrete, pink, scaly plaques clinically suspicious of psoriasis were scattered diffusely on the arms and legs (Figure 1A). The patient denied arthralgias. Dupilumab was held, but the rash continued to spread on the arms and legs and was mildly ameliorated by topical 0.05% clobetasol ointment. Punch biopsy of a lesion on the right anterior thigh revealed an unusual combination of psoriasiform, spongiotic, and lichenoid dermatitis (Figure 2A). The patient was trialed on 10 mg daily acitretin for its non-immunosuppressive properties and cited efficacy in Grover's disease [2], lichenoid [3] eruptions, and psoriasiform [4] eruptions, however, with little improvement (Figure 1B). Treatment with 10 mg daily apremilast for its similar non-immunosuppressive properties and reported effect on Grover's disease [5] for one month yielded improvement.

Case 2

A 63-year-old female with stage IV colorectal cancer presented with a one-week history of pruritic rash and nail abnormalities. At the time of presentation, the patient's cancer was treated with 200 mg IV pembrolizumab every three weeks and TAK-981, a small molecule inhibitor of the small ubiquitin-related modifier protein pathway implicated in cancer pathogenesis. Clinical examination revealed diffuse pink, scaly papules and plaques on the buttocks and back, demonstrating a koebner phenomenon over a scar on her knee. The total body surface area (BSA) was 10% (Figure 1C-1D), with bilateral distal nail yellowing and splinter hemorrhages (Figure 1E). The patient denied arthralgias. Given clinical suspicion for psoriasis, triamcinolone 0.1% ointment twice a day with halobetasol 0.05% ointment for thicker areas was prescribed. However, follow-up one week later revealed a spread of the rash with scattered pink scaly papules and plaques covering a total BSA of 10-30% with increased distal nail yellowing, splinter hemorrhages, and hyperkeratosis of the bilateral fingertips. Due to progressing symptoms, cancer treatment was held for one week. Given the psoriasiform clinical and morphologic characteristics of the rash, the patient started treatment with ixekizumab.

Interestingly, a punch biopsy of a lesion on the left lower back revealed a similar combination of psoriasiform, spongiotic, and lichenoid dermatitis (Figure 2B). Follow-up two weeks after starting ixekizumab treatment revealed improvement in symptoms and BSA decrease to 5% even after resumption of her cancer therapy.

## Discussion

ICIs are monoclonal antibodies that target coregulatory molecules like programmed cell death protein 1 (PD-1) to promote an immune response to target malignancy through T-cell activation [1]. Unfortunately, this novel class of immunotherapeutics can commonly cause irAEs and systemic toxicities from immune system activation by the ICI [1].

Skin toxicities are the most commonly reported irAEs [1] and ICI-induced psoriasiform rash, either de novo or reactivated, is well-documented in the literature [6,7]. Both pembrolizumab and nivolumab target the PD-1 receptor to upregulate immune response against malignancy and have been reported to cause psoriasiform eruptions [6]. Eruption correlates strongly with tumor response [6]. Skin biopsy specimens reveal features of psoriasis vulgaris, such as parakeratosis, hypogranulosis, acanthosis with elongation of rete ridges, and a perivascular lymphocytic infiltration [6]. In patients with nivolumab-induced [8,9] and pembrolizumab-induced [10] psoriasiform rash, histopathology may show spongiosis in addition to classical psoriasis features. Treatment for ICI-induced psoriasiform rash includes high-potency topical corticosteroids, vitamin D3 analogs, narrowband ultraviolet B phototherapy, and retinoids or biologics [6].

We report a novel ICI-induced clinicopathological entity with unusual combined psoriasiform, spongiotic, and lichenoid dermatitis on histopathology. It was reported previously only in association with antitumor necrosis factor (TNF)-alpha treatment [11]. In case 1, a direct relationship between the onset of characteristic skin lesions and ICI initiation was observed. While reports show dupilumab-induced psoriasis in atopic dermatitis patients, it is unlikely that dupilumab provoked our patient's eruption as discontinuation of the medication did not lead to rash resolution. In case 2, rash onset and pembrolizumab administration were similarly correlated, indicating that both patients' cutaneous eruptions were likely ICI-induced.

Our report also suggests that therapeutics for psoriasis are effective in treating ICI-induced psoriasiform rash with histopathological findings of psoriasiform, spongiotic, and lichenoid dermatitis. While acitretin and apremilast were selected for treatment in case 1, given separate reports of their cited efficacy in both psoriasis and Grover's disease, ICI-induced psoriasiform rash showed the most rapid and notable improvement after targeted treatment with ixekizumab, a selective inhibitor of the pro-inflammatory cytokine, IL-17A, which plays a key role in psoriasis pathogenesis. Early data indicate that IL-17A inhibitors may be safely used in cancer patients [12].

## Conclusions

To our knowledge, we report the first two cases of ICI-induced psoriasiform eruptions with distinct histopathological findings of psoriasiform, spongiotic, and lichenoid dermatitis. Our cases add to the literature on reported immune toxicities related to ICI use and illustrate the utility of targeted immune suppression of pathways in disease phenotype to allow for ICI continuation and maximizing ICI cytotoxic effect for optimal cancer treatment outcomes. However, further research is needed to study the pathogenesis and underlying molecular characteristics of this novel clinicopathologic entity.
